# Editorial: Computational Proteomics and Integration of Data Resources for Advanced Studies in Life Sciences

**DOI:** 10.3389/fgene.2021.729013

**Published:** 2021-07-23

**Authors:** Anna Marabotti, Paolo Romano, Angelo Facchiano

**Affiliations:** ^1^Department of Chemistry and Biology “A. Zambelli” University of Salerno, Fisciano, Italy; ^2^Proteomics and Mass Spectrometry, IRCCS Ospedale Policlinico San Martino, Genova, Italy; ^3^National Research Council, Institute of Food Science, CNR-ISA, Avellino, Italy

**Keywords:** proteomics, bioinformatics, computational proteomics, web tools and applications, data analysis

Proteomics is one of the omics techniques with expanding applications in biological and medical studies, aimed at protein identification and quantification, protein-protein interaction, structure, and dynamics characterization. The heterogeneity of data produced increases the complexity of the data analysis required. Computational and bioinformatics resources offer tools for the needed integration of data, by considering that the full interpretation of complex phenomena now requires comparison and integration of knowledge from different levels of genomics (gene expression, protein expression, and protein function), in the biomedical area as well as in biotechnology and agri-food studies.

Following the NETTAB/BBCC2019 Conference (http://www.igst.it/nettab/2019/), this Research Topic has been aimed to collect articles that describe development of novel tools for the analysis of proteomics data and their integration with omics data from genomics level in studies that add knowledge into the complexity of biology using inter-omics approaches. The Conference has been organized as a joint meeting of two annual events, NETTAB (http://www.nettab.org) and BBCC (http://www.bbcc-meetings.it), both followed by the publication of article collections in specialized journals (Armano et al., 2007; Romano et al., 2011, 2017, 2019; Marabotti et al., 2018; Chicco et al., 2020; Facchiano, 2020). The Conference has been attended by about 80 researchers, hosted a special session on “Computational Proteomics” and a panel discussion entitled “Community efforts for computational proteomics”, co-chaired by the leaders of the ELIXIR Proteomics Community, Oliver Kohlbacher (University of Tübingen), Lennart Martens (VIB-UGent Center for Medical Biotechnology) and Juan Antonio Vizcaíno (EBI).

## The Article Collection

The Research Topic is a collection of six articles, five of which are “Original Research” articles, and one is a “Technology and Code” article.

a) Original Research Articles.

In the article entitled “FGF2 affects Parkinson's disease-associated molecular networks through exosomal Rab8b/Rab31” (Kumar R. et al.), the Authors performed a bioinformatics analysis to dissect the interactions of two exosomal members of Rab family, i.e., Rab8b and Rab31 (upregulated by FGF2 stimulation in cultured hippocampal neurons), by means of a network analysis approach. In this way, they identified several proteins associated with Parkinson's disease in cellular components of both the central and the enteric nervous system. This work revealed previously unknown insights into changes associated with this disease. This approach can be applied to the study of disease progression in complex diseases such as neurodegenerative conditions.

The article “Gene Set Enrichment Analysis of interaction networks weighted by node centrality” (Zito et al.) presents a method to perform Gene Set Enrichment Analysis (GSEA) based on the topological parameter betweenness centrality of protein-protein interaction (PPI) networks, with the aim of applying GSEA to data sets and studies in the absence of gene expression data. Integration of data from different sources has been applied in two examples, thus proposing the approach can be applied as a tool for precision medicine and network medicine studies.

In the article “*In vivo* cross-linking MS of the complement system MAC assembled on live Gram-positive bacteria” (Khakzad et al.), the Authors studied the structure of the multicomponent human complement system membrane attack complex (MAC) by combining *in vivo* cross-linking mass spectrometry with computational macromolecular modeling and docking. They developed an affinity procedure followed by chemical cross-linking on human blood plasma, which allowed to obtain a quaternary model of the assembled MAC in its native environment, also supported by experimental models based on existing X-ray crystallography and electron cryo-microscopy.

In the article “An integrative computational approach based on expression similarity signatures to identify protein-protein interaction networks in female-specific cancers” (Pane et al.) the Authors developed an integrative computational approach based on gene expression similarities and PPI network analysis to highlight the pivotal and potential estrogen-dependent molecular mechanisms promoting carcinogenesis, identifying and extrapolating the common hubs in three female-specific cancers (breast, ovarian, and endometrial cancers). This study represents a well-designed multidisciplinary investigation in this field, and may have important clinical implications toward precision medicine for the identification of novel targeted therapies and/or prognostic factors for the management of female hormone-dependent cancers.

The article entitled “Construction of unified human antimicrobial and immunomodulatory peptide database and examination of antimicrobial and immunomodulatory peptides in Alzheimer's disease (AD) using network analysis of proteomics datasets” (Kumar A. et al.) present a workflow for the utilization of the publicly accessible proteomics datasets deposited in the ProteomeXchange resource (http://www.proteomexchange.org/), aimed to analyze the network of antimicrobial and immunomodulatory peptides (AMPs) in AD. The Authors created a database collecting AMPs, then examined by Cytoscape and different plugins the AMP interaction networks characteristic for AD, by using protein-protein and gene interaction data.

b) Technology and Code Article

In “GeenaR: a web tool for reproducible MALDI-TOF analysis” (Del Prete et al.), the Authors present a portal for the analysis of MALDI-TOF spectra (http://proteomics.hsanmartino.it/geenar/). GeenaR allows to pre-process spectra, through various methods and algorithms, and to compare them to identify molecules with potential as biomarkers. GeenaR interface gathers spectra and requested analyses and launches a script that runs analyses and returns results along with the R code used. The strength and innovation of the system is that, due to its modularity, it is flexible and easy to extend by adding new methods. Moreover, by returning the code it supports reproducible analysis.

## Discussion

The variety of topics covered in the articles present in the Research Topic (and even more in the contributions presented during the meeting) confirms the vast possibility of application of computational methods in proteomics. The panel discussion in the Conference Program highlighted the importance of making scientists aware that proteomics data can complement the “static view” of the genome by adding a richer set of information. On the other hand, the proteome is more complex and variable than the genome and the transcriptome, and data are more heterogeneous, therefore they are often harder to manage to extract useful information. Proteomics is evolving rapidly, and it is essential that “wet” biologists make a correct use of the bioinformatics tools available for proteomics data, to avoid errors and misinterpretations. Moreover, proteomics must gain further visibility among the scientists, because a biological system cannot be fully understood without taking it into account. To do so, it will be important to organize training sessions and events to bring “wet” and “*in silico*” scientists together for an exchange of ideas and to improve expertise, as well as to promote the importance of applying different computational approaches for the study of biomedical subjects at a proteomics level, as it was shown during the meeting.

## Author Contributions

All authors listed have made a substantial, direct and intellectual contribution to the work, and approved it for publication.

## Conflict of Interest

The authors declare that the research was conducted in the absence of any commercial or financial relationships that could be construed as a potential conflict of interest.

## Publisher's Note

All claims expressed in this article are solely those of the authors and do not necessarily represent those of their affiliated organizations, or those of the publisher, the editors and the reviewers. Any product that may be evaluated in this article, or claim that may be made by its manufacturer, is not guaranteed or endorsed by the publisher.
